# Differential regulation of phosphorylation, structure, and stability of circadian clock protein FRQ isoforms

**DOI:** 10.1016/j.jbc.2023.104597

**Published:** 2023-03-09

**Authors:** Xianyun Chen, Xiaolan Liu, Xihui Gan, Silin Li, Huan Ma, Lin Zhang, Peiliang Wang, Yunzhen Li, Tianyu Huang, Xiaolin Yang, Ling Fang, Yingying Liang, Jingjing Wu, Tongyue Chen, Zengxuan Zhou, Xiao Liu, Jinhu Guo

**Affiliations:** 1 State Key Laboratory of Biocontrol, Key Laboratory of Gene Engineering of the Ministry of Education, School of Life Sciences, Sun Yat-sen University, Guangzhou, China; 2 State Key Laboratory of Mycology, Institute of Microbiology, Chinese Academy of Sciences, Beijing, China; 3 Sun Yat-sen University Instrumental Analysis & Research Center, Sun Yat-sen University, Guangzhou, China

**Keywords:** circadian clock, *Neurospora crassa*, FREQUENCY (FRQ), phosphorylation, conformation

## Abstract

*Neurospora crassa* is an important model organism for circadian clock research. The *Neurospora* core circadian component FRQ protein has two isoforms, large FRQ (l-FRQ) and small FRQ (s-FRQ), of which l-FRQ bears an additional N-terminal 99-amino acid fragment. However, how the FRQ isoforms operate differentially in regulating the circadian clock remains elusive. Here, we show l-FRQ and s-FRQ play different roles in regulating the circadian negative feedback loop. Compared to s-FRQ, l-FRQ is less stable and undergoes hypophosphorylation and faster degradation. The phosphorylation of the C-terminal l-FRQ 794-aa fragment was markedly higher than that of s-FRQ, suggesting the l-FRQ N-terminal 99-aa region may regulate the phosphorylation of the entire FRQ protein. Quantitative label-free LC/MS analysis identified several peptides that were differentially phosphorylated between l-FRQ and s-FRQ, which were distributed in FRQ in an interlaced fashion. Furthermore, we identified two novel phosphorylation sites, S765 and T781; mutations S765A and T781A showed no significant effects on conidiation rhythmicity, although T781 conferred FRQ stability. These findings demonstrate that FRQ isoforms play differential roles in the circadian negative feedback loop and undergo different regulations of phosphorylation, structure, and stability. The l-FRQ N-terminal 99-aa region plays an important role in regulating the phosphorylation, stability, conformation, and function of the FRQ protein. As the FRQ circadian clock counterparts in other species also have isoforms or paralogues, these findings will also further our understanding of the underlying regulatory mechanisms of the circadian clock in other organisms based on the high conservation of circadian clocks in eukaryotes.

Circadian clocks enable organisms to predict and adjust their physiology and behavior to the daily changing environment (1). It has been demonstrated that misalignment in circadian rhythms leads to maladaptation to the daily alternating environment in many species (2). In humans, the circadian clock is considered one of the most important hallmarks of health, and circadian misalignment causes a variety of diseases (3).

The circadian clock is finely regulated at the molecular level. The filamentous fungus *Neurospora crassa* is a classical model for circadian research, which has substantially contributed to the understanding of circadian systems in other organisms (4). In *Neurospora*, White Collar 1 (WC-1) and WC-2 are the two positive elements that form the White Collar Complex (WCC) to activate the transcription of the *frequency* (*frq*) gene; and the latter encodes the FRQ protein which is the negative element (5, 6). FRQ is the negative component in the *Neurospora* circadian clock, which represses the function of WCC as transcription factors in the negative feedback loop and supports WCC levels in an interlocked positive limb (7, 8, 9). FRQ proteins form dimers *via* the coiled-coil domain that is critical for its circadian clock function (10). FRQ also recruits casein kinases to regulate the phosphorylation of WCC proteins (11). Hyperphosphorylated WCC proteins are stable and inactive in transcription (12, 13, 14). Phosphatase 2A dephosphorylates and reactivates WCC by countering the function of casein kinases (11, 12, 13, 14, 15).

Dynamic phosphorylation of circadian clock proteins is highly conserved in eukaryotes (16). In *Neurospora*, 101 *in vivo*/*in vitro* phosphorylation sites have been identified in FRQ, and it is very likely that FRQ may harbor even more phosphorylation sites (17, 18). The phosphorylation of FRQ is dynamically controlled by a number of protein kinases including casein kinas 1 (CK1), CK2, checkpoint kinase 2, calcium/calmodulin-dependent kinase-1, Protein kinase A (PKA), in which FRQ-bound CK1a plays multiple roles in the circadian negative feedback loop (17, 18, 19). The degradation rate of FRQ is primarily mediated by the affinity of FRQ–CK1 interaction, which roughly correlates with the circadian period (20).

The open reading frame (ORF) of *frq* contains three initiation codons, and the second codon is rarely used (21). Translation from the first initiation codon results in the synthesis of a protein product with 989 amino acid residues (aa), which is called large FRQ (l-FRQ). *Frq* also possesses up to six introns that undergo complex control of alternative splicing, producing eight splicing variants in total (21, 22, 23, 24). Removal of the sixth intron (I-6) in *frq* pre-mRNA by alternative splicing eliminates the translation initiation from the first codon, which results in the synthesis of a protein isoform lacking the N-terminal 99 aa (N-99), which is called small FRQ (s-FRQ) (22, 23). Several splicing factors or regulators, for example, PRP5, PRMT5, and the snRNA *U4-2*, *U5*, are implicated in the regulation of *frq* splicing and/or circadian rhythms. Complexes for mRNA processing and surveillance, including the exosome and nonsense-mediated RNA decay machinery, have been demonstrated to regulate the splicing and decay of transcript variants (25, 26, 27). The *frq* gene has several untranslated ORFs in its 5′ untranslated region, which regulates the temperature sensitivity during FRQ translation (21, 22, 23).

The synthesis ratio of l-FRQ/s-FRQ changes according to the ambient temperature. Each of the FRQ isoforms can independently sustain rhythmicity at certain temperatures although synergetic expression of both is critical for fine tuning of clock (21, 23, 28). The strain exclusively expressing l-FRQ (*l-frq* strain) displays a shorter period while the strain expressing only s-FRQ (*s-frq* strain) displays a longer period, in comparison to the wild type, respectively. S-FRQ loses its function at high temperatures, while l-FRQ becomes dysfunctional at low temperatures (21), suggesting that both s-FRQ and l-FRQ are required for clock and carry out differential circadian functions. A significantly higher proportion of s-FRQ is localized in the nucleus than l-FRQ (29), also suggesting the differences in phosphorylation and function between s-FRQ and l-FRQ. In addition, l-FRQ is more effective in promoting the day/night growth ratio (26). However, little is known about how FRQ isoforms operate differentially in regulating the circadian clock.

In this work, we demonstrate the different roles of l-FRQ and s-FRQ and the underlying mechanisms in the regulation of the circadian clock. The N-99 aa fragment of l-FRQ plays an important role in controlling the phosphorylation, stability, conformation, and function of FRQ. In addition, we identified two novel FRQ phosphorylation sites and characterized their effects on circadian rhythms.

## Results

### l-FRQ and s-FRQ act differently in circadian feedback loops

Both splicing and ambient temperature contribute to the production of s-FRQ and l-FRQ (22, 23, 27). To validate whether alternative splicing directly regulates the ratio of FRQ isoforms, we treated the *mdr3*^*KO*^ strain with spliceostatin A (SSA), an inhibitor of spliceosome assembly (30). Multiple drug resistance 3 (MDR3) is a protein responsible for drug metabolism (31); therefore, the lack of MDR3 in the *mdr3*^*KO*^ strain could prevent its potential resistance against SSA. The results from dephosphorylation experiments showed that the samples in the presence of SSA showed a lower proportion of s-FRQ than the untreated samples in the *mdr3*^*KO*^ strain (Fig. S1*A*), indicating that suppression of the splicing of *frq* I-6 directly affects the production of the synthesis ratio of FRQ isoforms.

**Figure 1:**
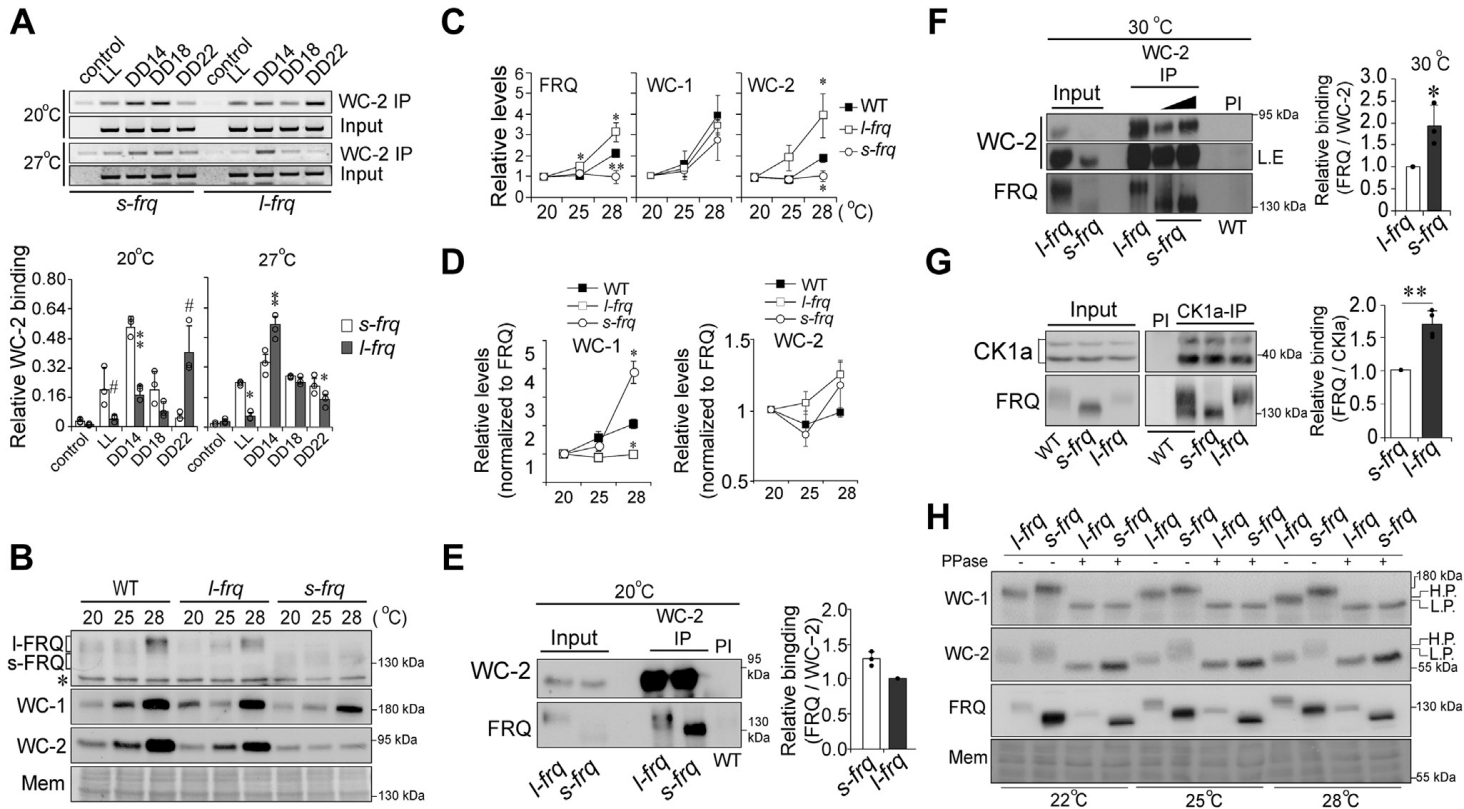
**s-FRQ and l-FRQ differentially regulate the negative feedback loop of the circadian clock.***A*, ChIP assay results showing differential binding of WCC to *frq* promoters in *s-frq* and *l-frq* strains (*top*) and statistical results (*bottom*). Sampling was carried out at the indicated time points. DD denotes constant darkness and LL denotes constant light. *B*, Western blot results showing the effects of s-FRQ and l-FRQ on WCC protein levels at three temperatures. *C*, statistics of relative protein levels of FRQ, WC-1, and WC-2 according to the results from (*B*). *D*, statistics of relative protein levels of WC-1 and WC-2 normalized to FRQ. The expression levels of FRQ, WC-1 and WC-2 were according to the results from (*B*). *E* and *F*, Co-IP results showing the binding of WC-2 to s-FRQ and l-FRQ at 20 °C (*E*) and 30 °C (*F*). Statistical results are shown on the *right*. Samples treated with PI were included as controls. Long exposure of WC-2 Western blot is shown. In (*E*) one- and twofold of total protein from the *s-frq* strain were loaded for comparison. *G*, Co-IP results showing the binding of CK1a to s-FRQ and l-FRQ at 25 °C. Statistical results are shown on the *right*. An antibody against CK1a was used to conduct the immunoprecipitation. Samples treated with preimmune serum (PI) were included as controls. *H*, expression and phosphorylation of WC-1 and WC-2 in *l-frq* and *s-frq* strains at 22 °C, 25 °C, and 28 °C. The ratio of acrylamide–bisacrylamide in the gel was 149:1 and the concentration was 10%. Values are the mean ± SD (n = 3). ∗*p* < 0.05, ∗∗*p* < 0.01 and ^#^*p* < 0.001. ChIP, Chromatin immunoprecipitation; CHX, cycloheximide; CO-IP, co-immunoprecipitation; *frq*, *frequency*; l-FRQ, large FRQ; PI, preimmune serum; s-FRQ, small FRQ; WC-1, White Collar 1.

WC-2 binds to WC-1 to form the WCC complex that binds to the promoters of *frq* and other clock-controlled genes in a circadian fashion, and FRQ represses the function of WCC in a negative feedback loop (5, 7). To compare the functional difference between FRQ isoforms in the circadian negative feedback loop, we conducted chromatin immunoprecipitation (ChIP) assays with WC-2 antibody to assess the binding efficiency of WCC to the *frq* promoter in the *l*-*frq* and *s-frq* strains at 20 °C and 27 °C. In the results at 20 °C, WC-2 showed the highest binding to *frq* promoter at DD14 in the *s-frq* strain and DD22 in the *l-frq* strain; WC-2 showed the highest binding to *frq* promoter at DD14 in both *s-frq* and *l-frq* strains but lower binding at LL, DD18, and DD22 in *l-frq* strain at 27 °C (Fig. 1*A*). These data support that l-FRQ and s-FRQ may function differentially to sustain circadian rhythmicity in the negative feedback loop in a temperature-sensitive manner (21).

As the ratio of FRQ isoforms differs at high and low temperatures, changed s-FRQ/l-FRQ ratios may affect the levels of WCC proteins (21, 23, 28). We next performed Western blot analysis to examine the levels of the clock proteins FRQ, WC-1 and WC-2 in *l-frq*, *s-frq*, and WT strains at three temperatures, which were chosen according to the previous reports (21, 23, 28). The results showed that with increasing temperature, the levels of both WC-2 and FRQ were significantly induced in the *l-frq* strain. By contrast, the change in FRQ and WC-2 was marginal in the *s-frq* strain. In *kaj120*, a rescued strain expressing both s-FRQ and l-FRQ in the background of the *frq* knockout strain (*frq*^*10*^), the levels of WC-2 and FRQ were between those of *s-frq* and *l-frq*, reflecting a mixed effect. By contrast, the change in WC-1 levels was comparable in all three strains (Fig. 1, *B* and *C*), which is in agreement with previous reports (21, 23). However, when considering the levels of FRQ in different strains, it is noteworthy that the amount of WC-1 but not WC-2 in the *s-frq* strain was much higher than that in the other two strains at 28 °C (Fig. 1*D*).

Besides its role in the circadian negative feedback loop, FRQ also supports the transcription of *wc-2* and the accumulation of WC-1 protein through different positive limbs (8, 9, 32). To address the different expressions of WC-1 and WC-2, we checked the mRNA levels of *wc-1* and *wc-2* which showed comparable expression at both 20 °C and 28 °C (Fig. S1*B*). WC-2 protein showed a faster degradation rate at 28 °C than that at 20 °C, while the degradation rates of WC-1 or WC-2 were comparable in *s-frq* or *l-frq* strains at both 20 °C and 28 °C (Fig. S1, *C*–*F*). Therefore, together, these facts suggest that the increased degradation of WC-2 at 28 °C in *s-frq* may account for the difference in WC-2 expression from that in *l-frq*.

In addition to the repression of WCC function in promoting transcription, FRQ also facilitates the phosphorylation and accumulation of WCC which constitutes a positive loop in the circadian clock negative feedback circuits, which leads to reduced WCC turnover. Therefore, we measured the levels of WCC proteins as this way the function of FRQ could be determined (8, 14). And the results suggest that s-FRQ is more efficient in promoting the level of WC-1 at higher temperatures.

FRQ binds to the WCC complex to repress its own transcription, which closes the negative feedback loop. Next, we conducted co-immunoprecipitation (CO-IP) with WC-2 antiserum in the *l-frq* and *s-frq* strains to assess the association between WCC and s-FRQ and l-FRQ at 20 °C and 30 °C, respectively. The CO-IP results showed that s-FRQ bound to more WCC than l-FRQ at both temperatures (Fig. 1, *E* and *F*), which suggests that s-FRQ exhibits higher binding to WCC in the negative feedback loop.

The FRQ-CK1a association determines the prevalent phosphorylation of FRQ and the circadian period (20, 33). Compared to l-FRQ, the ratio of s-FRQ bound to CK1a was significantly lower than l-FRQ and wild type (Fig. 1*G*), suggesting that the phosphorylation of FRQ isoforms is differentially regulated by CK1a. WCC proteins undergo phosphorylation and dephosphorylation by related kinases and phosphatases, and the WCC phosphorylation levels determine their turnover (19). We conducted λ phosphatase treatment and Western blot analysis to investigate the expression, and surprisingly, phosphorylation of WCC proteins in the *l-frq* and *s-frq* strains and found that both WC-1 and WC-2 were hyperphosphorylated in *s-frq* compared to that in *l-frq* (Fig. 1*H*).

### l-FRQ shows faster phosphorylation, turnover, and it possesses a looser structure compared to s-FRQ

**Figure 2:**
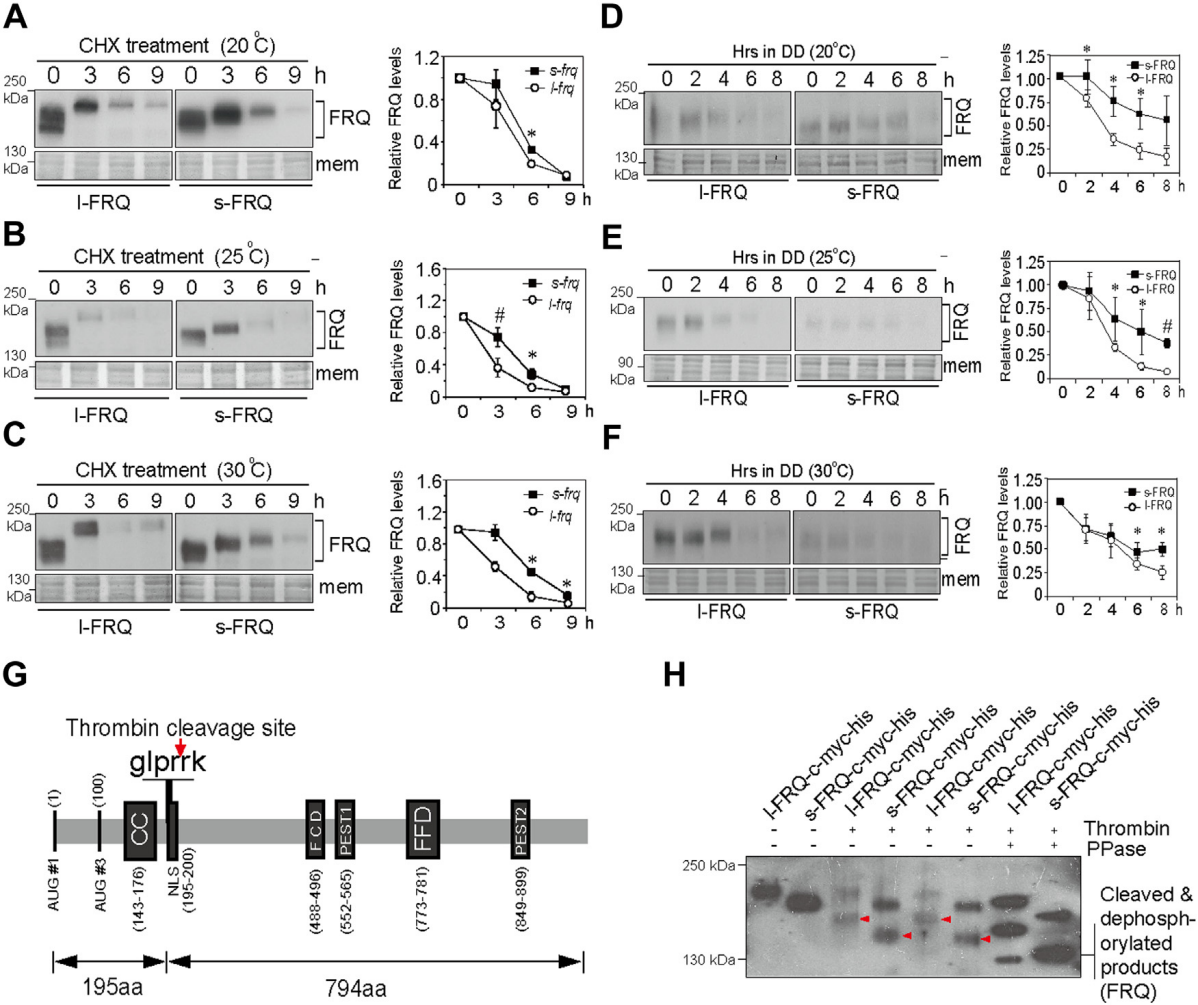
**l-FRQ is more rapidly phosphorylated and degraded than s-FRQ.***A*–*C*, Western blot results showing degradation of s-FRQ and l-FRQ after CHX treatment (10 μg/ml) at three temperatures. For comparison, approximately two times the protein extracts from the *s-frq* strain were loaded. *D*–*F*, Western blot results showing phosphorylation of l-FRQ and s-FRQ proteins after the transition from LL to DD at three temperatures. The relative phosphorylation levels were estimated by the mobility rates in electrophoresis. Higher mass FRQ proteins move slowly due to hyperphosphorylation, while lower-mass FRQ proteins move quickly. For easier comparison of the phosphorylation states, the s-FRQ samples were loaded for electrophoresis earlier than the l-FRQ samples to make them run at comparable horizontal levels. *G*, schematic of the l-FRQ structure and phosphorylation sites. The *arrow points* to the thrombin cleavage site. The thrombin cleavage site is also present in s-FRQ. *H*, Western blot results of the thrombin cleavage assay with MYC antibody. Representative results from three independent experiments are shown. *Red dots* indicate the intermediate products of uncleaved or non-phosphorylated proteins and *red arrows* indicate the cleaved and dephosphorylated products. Values are the mean ± SD (n = 3). ∗*p* < 0.05 and ∗∗*p* < 0.01, ^#^*p* < 0.001. CC, Coiled-coil domain; CHX, cycloheximide; FFD, FRQ–FRH interaction domain; *frq*, *frequency*; NLS, nuclear localization signal; FCD, FRQ-CKI interacting domain; PEST, polypeptide sequences enriched in proline (P), glutamic acid (E), serine (S) and threonine (T) that target proteins for rapid destruction.

Phosphorylation and stability are two crucial aspects of FRQ protein, which are closely related to its function (17, 34). However, the difference in phosphorylation, structure, and degradation between s-FRQ and l-FRQ remains elusive. To this end, we assessed the difference in the degradation rates between l-FRQ and s-FRQ by cycloheximide (CHX) treatment. CHX is a translation inhibitor that allows us to measure the turnover rate of proteins of interest. The Western blotting results showed that the degradation of l-FRQ was faster than s-FRQ at all of the three tested temperatures (20 °C, 25 °C, and 30 °C) (Fig. 2, *A*–*C*). To exclude the possibility of artificial effects caused by CHX, we also examined the degradation rates of l-FRQ and s-FRQ by LD transition experiments. FRQ protein maintains a relatively high level in constant light and undergoes gradual degradation in the first several hours after transition into darkness, and the decrease in l-FRQ level was faster at 20 °C and 25 °C (Fig. 2, *D*–*F*).

We also compared the changes in phosphorylation levels of l-FRQ and s-FRQ during their degradation. The CHX-treated protein samples of *l-frq* were loaded for electrophoresis for approximately 10 min, and then the CHX-treated protein samples of *s-frq* were loaded on the same gel, to allow the samples of *s-frq* and *l-frq* to run at comparable levels for better comparison of their phosphorylation. The phosphorylation of l-FRQ reached its highest level after 3 h while the phosphorylation of s-FRQ was significantly delayed (Fig. S2, *A*–*C*). These results demonstrate the differential phosphorylation and turnover between the FRQ isoforms.

As the entire sequence of s-FRQ is identical to the C-terminus of l-FRQ (100 aa–989 aa), the higher phosphorylation level of l-FRQ might be attributed to higher phosphorylation of the 890 aa C-terminus. Another possibility is that the l-FRQ N-99 fragment itself but not the rest part is more phosphorylated in l-FRQ. A third possibility is the combination of both.

Sequence analysis indicates that both l-FRQ and s-FRQ harbor one common thrombin cleavage site, which is located between R195 and R196 in l-FRQ and between R96 and R97 in s-FRQ (Fig. 2*G*). The constructs expressing l-FRQ-c-Myc-His and s-FRQ-c-Myc-His bearing were transformed into the *frq*^*10*^ strain, respectively, and the resultant transformants *frq10*, *s-frq-c-myc-his*, and *frq10*, *l-frq-c-myc-his* were obtained. The l-FRQ-c-Myc-His and s-FRQ-c-Myc-His proteins were purified and subjected to thrombin cleavage and λ-phosphatase for dephosphorylation. The migration of cleaved products was obviously slower in l-FRQ-c-Myc-His than that in s-FRQ-c-Myc-His (Fig. 2*H*), suggesting that at least the C-terminus of FRQ is more hyperphosphorylated in l-FRQ than that in s-FRQ and the N-99 aa fragment of l-FRQ regulates the phosphorylation of FRQ C-terminus.

**Figure 3:**
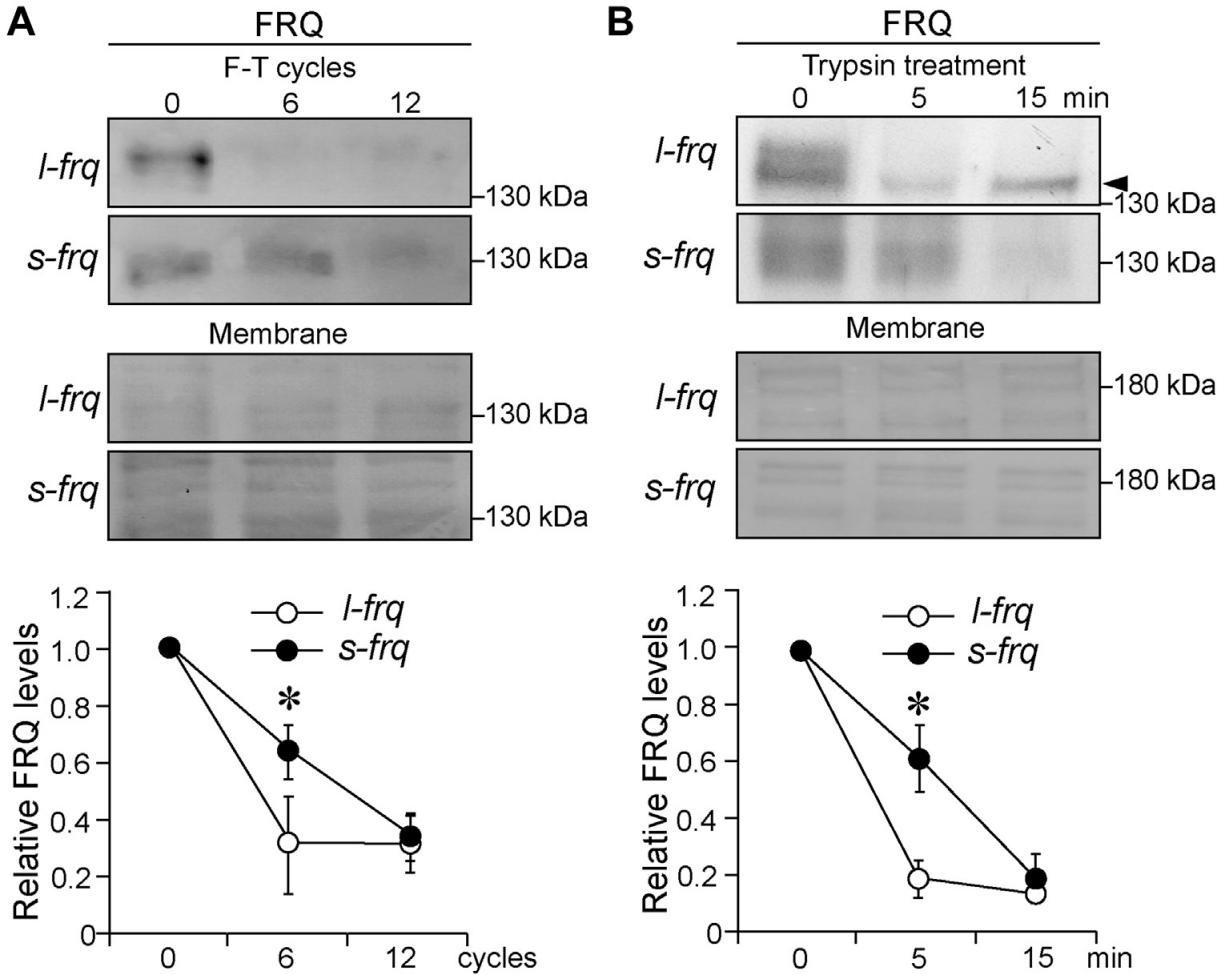
l**-FRQ is less stable and more sensitive to trypsin digestion than s-FRQ.***A* and *B*, Western blot results showing the sensitivity of s-FRQ and l-FRQ to F-T cycles (*A*) and trypsin (0.5 μg/ml) digestion (*B*). *Arrow* denotes non-specific bands. Densitometric analysis results are shown. Values are the mean ± SD (n = 3). ∗*p* < 0.05 and ∗∗*p* < 0.01. *frq*, *frequency*; F-T, freeze-thaw; l-FRQ, large FRQ; s-FRQ, small FRQ.

Phosphorylation and degradation of FRQ are tightly linked to its structure (17, 34). FRQ is very likely an unstructured protein, and its phosphorylation is very extensive and dynamic (35, 36), which complicates the analysis of its structure. To assess the difference in the structure of s-FRQ and l-FRQ, we performed freeze–thaw and limited trypsin digestion experiments, respectively. After treatment for up to 12 freeze–thaw cycles, l-FRQ levels decreased more rapidly than s-FRQ (Fig. 3*A*). Similarly, the results from partial trypsin digestion showed that l-FRQ decreased more rapidly than s-FRQ (Fig. 3*B*). Together, these results indicate that l-FRQ might possess a looser structure that is more subject to phosphorylation and degradation, and the differential conformation of FRQ isoforms might result from the N-99 aa region itself and/or the effects of N-99 aa region on the rest parts of FRQ.

### Mapping and analysis of the critical region in the l-FRQ N-terminus

**Figure 4:**
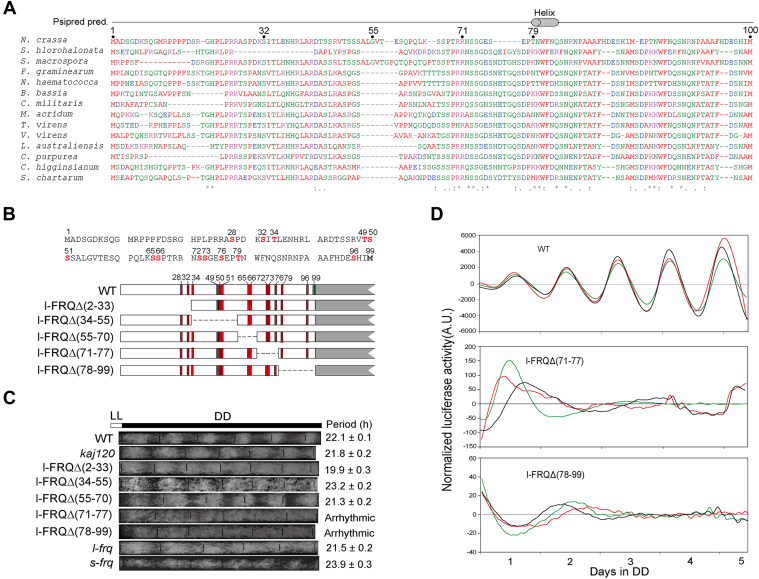
**Differential contribution of deletions in the N-terminus to the circadian clock function of l-FRQ.***A*, sequence alignment of the *N. crassa* FRQ 1 to 100 aa region with some other fungal FRQ homologs. *B*, schematic of the sequence of N-99 aa of l-FRQ and internal deletions in various mutants. The third initiation codon (M at 99 aa) was mutated in all the deletions. *C*, race tube assay of the deletion and mutation strains. The WT strain *301-5* strain was used as another control. Statistics of the periods of the deletion and mutation strains are shown. Values are the mean ± SD (n = 3). *D*, luciferase reporter assay showing *frq* promoter activity of the indicated strains in constant darkness. The measurement of luciferase activity was normalized to subtract the baseline luciferase signal. Results of representative three transformants are shown for each strain, denoted in different colors. A.U., arbitrary units; CK1, casein kinase 1; N-99, *frq*, *frequency*; N-terminal 99 aa.

To map the minimal region involved in the determination of the function of the N-99 region, we generated a series of constructs with deletions in the N-99 region, which were transformed into *frq*^*10*^ (Fig. 4, *A* and *B*). The results of the race tube assay showed that l-FRQΔ(71–77) and l-FRQΔ(78–99) displayed arrhythmic phenotypes while other strains displayed rhythms of conidial zonation. The period of l-FRQΔ(2–33) was shorter while l-FRQΔ(34–55) was longer than *l-frq* (Fig. 4*C*).

We next introduced a luciferase reporter construct that is under the control of the *frq* promoter into the l-FRQΔ(71–77) and l-FRQΔ(78–99) strains and found that, consistently, robust rhythmic luciferase activity was abolished in these two strains (Fig. 4*D*). These data suggest that the aa 71 to 99 region is required for the function of the N-99 region, and deletion of it may compromise the function of the entire FRQ protein. In contrast, the strains harboring mutations in this region, including the strain with mutation of S73A and the M2 strain with mutations of S72A, S73A and S76A, displayed conidiation rhythmicity (17, 18). In these strains, functional s-FRQ might restore circadian rhythmicity as the third initiation codon remains intact.

**Figure 5:**
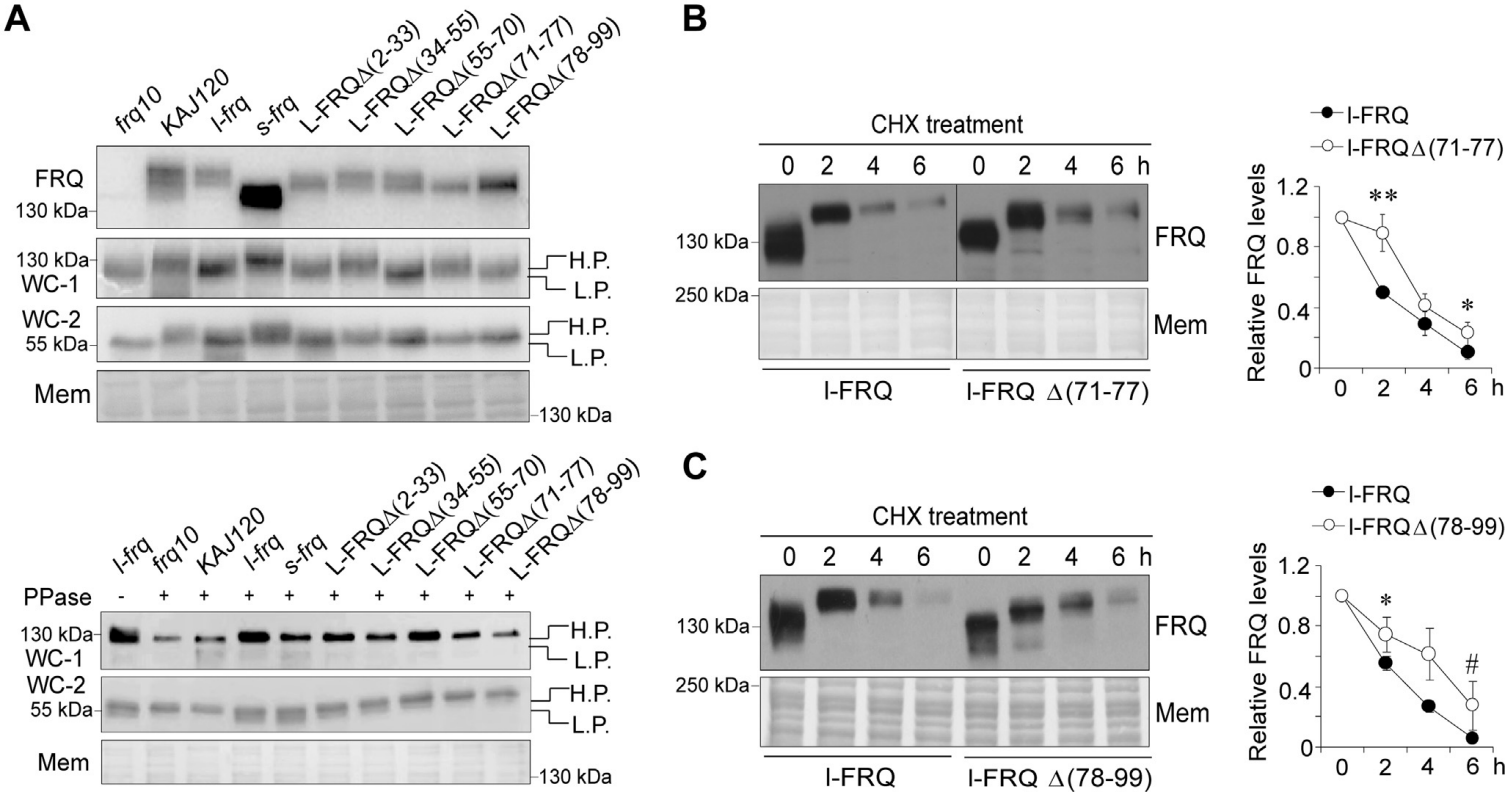
**FRQ 71 to 99 aa is critical for the function of l-FRQ.***A*, Western blot analysis of FRQ, WC-1, and WC-2 in *l-frq*, *s-frq*, kaj120, and FRQ deletion strains in the presence and absence of λ phosphatase. The ratio of acrylamide–bisacrylamide in the gel for analysis of WC-1 and WC-2 was 149:1 and the concentration was 10%. *B* and *C*, Western blot analysis of FRQ degradation in *l-frq*, FRQΔ(71–77) (*B*) FRQΔ(78–99) (*C*) strains. Densitometric analysis results are shown on the *right panels*. Samples were harvested at different time points after treatment with CHX. Values are the mean ± SD (n = 3). CHX, cycloheximide; *frq*, *frequency*; WC-1, White Collar 1.

We measured the expression and phosphorylation of clock proteins in the deletion strains, and the Western blotting results showed that deletion of l-FRQ(71–77) led to a dramatic decrease in the level of hyperphosphorylated FRQ proteins compared to other deletions (Fig. 5*A*). Deletion of these FRQ regions at its N terminus led to different influences on the phosphorylation of FRQ and WC-1. FRQ displayed overt hypophosphorylation in the strain l-FRQΔ(71–77) compared to other deletion strains; the WC-1 phosphorylation levels in l-FRQΔ(34–55) and l-FRQΔ(71–77) strains were higher than those in the strains of l-FRQΔ(2–33), l-FRQΔ(55–70) and l-FRQΔ(78–99). These data suggest that these regions differentially affect FRQ phosphorylation and contribute to the positive limb of the circadian clock (Fig. 5*A*). The turnover of l-FRQΔ(71–77) and l-FRQΔ(78–99) was significantly slower than that of l-FRQ (Fig. 5, *B* and *C*), suggesting that although these deletion strains cannot precisely mimic the phenotype of the *s-frq* strain, the 71 to 99 aa region plays a critical role in regulating FRQ function and circadian rhythms.

### Comparison of phosphorylated peptides between l-FRQ and s-FRQ

**Figure 6:**
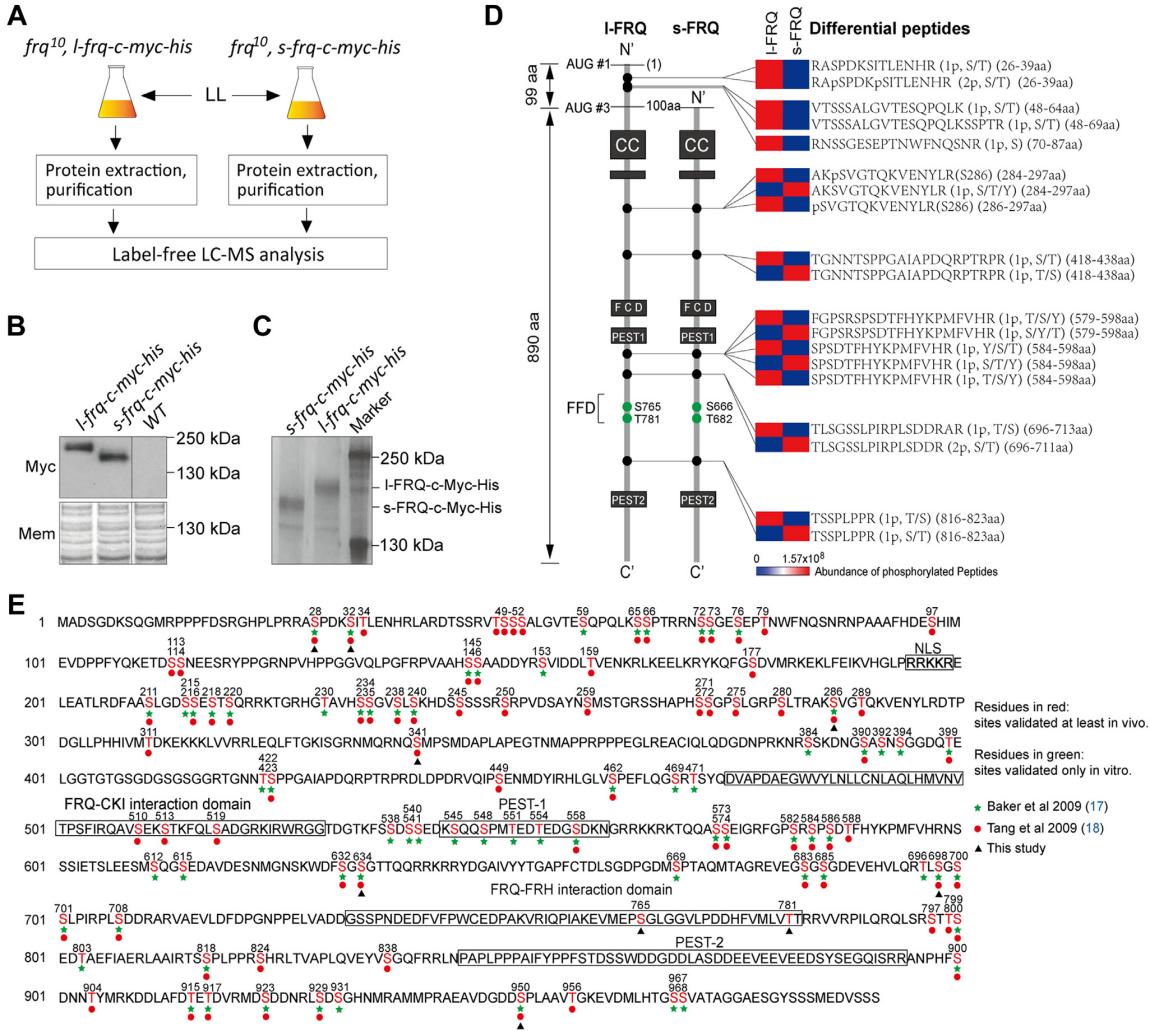
**Identification of phosphorylation sites on l/s-FRQ proteins.***A*, flowchart of the procedures of sample treatment and label-free LC-MS for quantitative analysis. *B*, Western blot showing the expression of FRQ in *frq*^*10*^, s-*frq*-c-myc-his and *frq*^*10*^, l-*frq*-c-myc-his strains. *C*, Coomassie blue-stained SDS/PAGE showing the purified FRQ-Myc-His from *frq*^*10*^, s-*frq*-c-myc-his and *frq*^*10*^, l-*frq*-c-myc-his strains. *D*, fragments showing differential phosphorylation between s-FRQ and l-FRQ. The numbers of phosphorylation sites and corresponding sites in each fragment are denoted. The phosphorylation data of these fragments were from the quantitative label-free LC/MS analysis. Two novel phosphorylation sites are labeled in *green*. The abundance represented by the heat map are averaged from three independent experiments. *frq*, *frequency*. *E*, map of FRQ phosphorylation sites. The sites identified in different studies are labeled in different colors.

As the phosphorylation level is higher in l-FRQ than that in s-FRQ, we conducted label-free LC-MS to quantitatively detect the phosphorylation sites in l/s-FRQ proteins. L-FRQ-c-Myc-His and s-FRQ-c-Myc-His proteins were purified from *frq*^*10*^, *l-frq-c-myc-his*, and *frq10*, *s-frq-c-myc-his* strains which were subsequently subjected to quantitative label-free LC-MS analysis (Fig. 6, *A*–*C*).

The results from label-free quantitative LC/MS analysis revealed 19 peptides with differential phosphorylation patterns between s-FRQ and l-FRQ. The preferential phosphorylation at the sites in the N-99 aa region supports the validity of the present analysis. In addition, phosphorylation occurred in s-FRQ and l-FRQ in an interlaced pattern, in which even some neighboring sites showed opposite preferential phosphorylation (Fig. 6*D* and Table S1). In addition, eight phosphorylation sites were identified, in which S28 and S32 are located in l-FRQ but not s-FRQ and S286 was prevalently phosphorylated in l-FRQ. Two novel sites (S765 and T781) were also identified and the phosphorylation of which was present in both s-FRQ and l-FRQ (Fig. 6, *D* and *E*).

### Identification and characterization of two novel phosphorylation sites on FRQ

**Figure 7:**
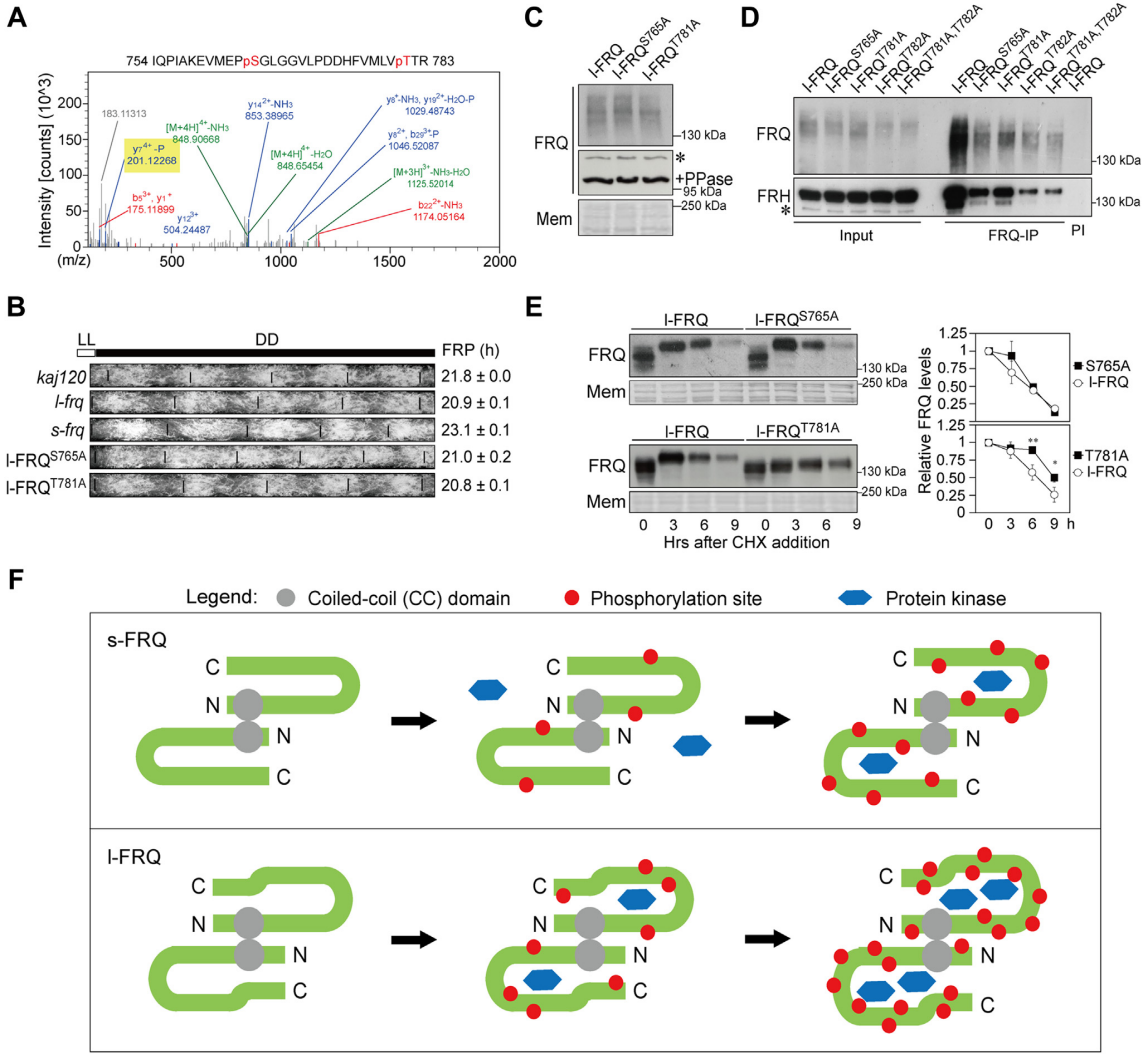
**Characterization of novel FRQ phosphorylation sites.***A*, MS spectrum of one FRQ phosphopeptide containing phosphorylation sites S765 and T781 in the purified FRQ sample. *B*, race tube assay showing the circadian conidiation rhythms of the indicated strains. Periods of circadian are denoted on the *right*. *Kaj120* was used as a control. Values are the mean ± SD (n = 3). *C*, protein levels and phosphorylation of FRQ in the mutants and *l-frq* strain. Samples were harvested in constant light. *Asterisk* indicates non-specific bands. *D*, Co-IP assays to assess the association between mutated l-FRQ proteins and FRH. Samples treated with PI were included as controls. *Asterisk* indicates non-specific bands. *E*, Western blots showing protein degradation after CHX treatment of FRQ^S765A^ and FRQ^T781A^. *Right*, densitometric analysis results are shown. Values are the mean ± SD (n = 3). ∗*p* < 0.05 and ∗∗*p* < 0.01. *F*, a model depicting hypothesized differential roles of s-FRQ and l-FRQ in regulating the circadian clock. FRQ proteins form dimers *via* the CC. The N-terminus (N) of FRQ is positively charged, while the C-terminus (C) is negatively charged. The *arrows* in gradient *green* indicate FRQ from N to C. *Black arrows* denote the transition of FRQ from hypophosphorylated status to hyperphosphorylated status. The diagram is not in scale and the phosphorylation sites are exaggerated for easier understanding. CC, coiled-coil domain; CHX, cycloheximide; CO-IP, co-immunoprecipitation; *frq*, *frequency*; PI, preimmune serum.

Previous studies have revealed that FRQ harbors up to 101 phosphorylation sites, which change in a circadian fashion (17, 18). In this work, the label-free quantitative LC/MS analysis identified two novel phosphorylation sites in FRQ, S765, and T781 (Figs. 6, *D* and *E* and 7*A*) which showed no significant difference in phosphorylation between s-FRQ and l-FRQ. Both of these sites are located in the FRQ6 region, and especially, T781 is located in the FRQ6B region which is critical for FRQ-FRH interaction and circadian rhythmicity (37, 38, 39).

We generated mutants harboring S765A (l-FRQ^S765A^) and T781A (l-FRQ^T781A^) in the l-*frq* strain, respectively. In addition, we also generated mutants of T782A (l-FRQ^T782A^) and T781A,T782A (l-FRQ^T781A,T782A^) although phosphorylation of T782 was not found in this work or in previous studies (17, 18). In race tube assay, the strains of l-FRQ^S765A^ and l-FRQ^T781A^ showed comparable conidiation periods to that of *l-frq* strain, a comparable conidiation period to l-*frq* (Fig. 7*B*). L-FRQ^T781A^ but not l-FRQ^S765A^ showed lower phosphorylation levels under constant light compared to that of l-FRQ (Figs. 7*C* and S3, *A* and *B*). Co-IP was conducted to measure the association between mutated FRQ and FRH, and the results showed that mutations of the three sites, S765A, T781A, and T782A, did not abolish the FRQ–FRH interaction (Fig. 7*D*). In most cases, a higher phosphorylation level is correlated with a decreased stability of FRQ (18), and consistently, the Western blotting results revealed that the T781A mutation stabilized FRQ while S765A showed no significant effects on FRQ turnover (Fig. 7*E*). The two mutations on s-FRQ, s-FRQ^S666A^ and s-FRQ^T682A^ which are counterparts to l-FRQ^S765A^ and l-FRQ^T781A^, respectively, showed no overt effects on conidiation rhythmicity, and mutation of s-FRQ^T682A^ but not s-FRQ^S666A^ caused a slower degradation rate compared to the *s-frq* strain (Fig. S4, *A*–*C*). The functional significances of phosphorylation on l-FRQ^T781^ and s-FRQ^T682^ remain to be further investigated.

## Discussion

The alternative splicing of *frq* I-6 results in the production of two FRQ isoforms, l-FRQ and s-FRQ (21, 22, 23). In this work, we show that s-FRQ more effectively promotes WC-1 expression at high temperatures (Fig. 1, *B*–*D*) and demonstrate that l-FRQ is more effective in recruiting CK1 which leads to its higher phosphorylation (Figs. 1*H* and 2*H*). L-FRQ shows faster phosphorylation and degradation than s-FRQ (Figs. 2, *A*–*H* and S2, *A*–*C*). All of these findings support that FRQ isoforms play differential roles in regulating *Neurospora* circadian rhythms.

The phosphorylation and degradation of FRQ are crucial for clock functions (40, 41, 42, 43). Usually, accelerated phosphorylation of FRQ which is determined by the FRQ-CK1 interaction tends to decrease its stability and leads to the shortening of circadian periods (5, 20, 40). The N-99 region of l-FRQ contains several conserved and dynamically phosphorylated sites, which are phosphorylated in the late time of a circadian day (17, 18). In l-FRQ, the N-terminal specific region has been proposed to promote the degradation of the entire protein, and extensive mutations of phosphorylation sites in the FRQ N-terminus led to decreased phosphorylation and increased stability in clock proteins, suggesting that phosphorylation of the N-terminus might be necessary for regulating FRQ turnover (17, 36). In this work, the thrombin cleavage results directly demonstrated that both of the N-99 region and the rest part of l-FRQ contribute to its different phosphorylation pattern from that of s-FRQ (Figs. 2*H* and 6*D*), which might contribute to the differential turnover, conformation and function between FRQ isoforms. Bake *et al.* reported that FRQ mutation S73 led to an increased period length (1.8 h–2.3 h) in the WT strain (18), which also suggests that in the N-99 region, this site may importantly contribute to the period difference between *s-frq* and *l-frq* strains.

Mutations of most phosphorylation sites in the middle part of FRQ lead to longer periods (17, 18). We identified that the phosphorylation pattern in some regions that contain multiple potential phosphorylation sites was different between s-FRQ and l-FRQ (Fig. 6*D*), which might be attributed to differential phosphorylation of the same sites or phosphorylation of different sites in the same regions. This interlaced phosphorylation pattern suggests that some of these phosphorylated regions may have distinct functions in controlling the circadian clock, and the N-terminal region of l-FRQ may play an important role in modulating phosphorylation, stability, conformation, and accessibility to kinases of the FRQ protein. Moreover, we identified two novel phosphorylation sites in the FRQ protein, S765 and T781 (Fig. 7*A*). Based on this report and previous reports (17, 18), 103 *in vivo* and *in vitro* phosphorylation sites have been found in FRQ in total (Fig. 6*E*). The very high abundance of phosphorylation sites indicates that FRQ has a very intricate structure that undergoes complex regulation.

The degradation rate of FRQ is regulated by CK1, which is negatively associated with *Neurospora* circadian period. Nonetheless, there are some exceptions, for example, in the strain lacking the F-box protein FWD-1, an E3 ubiquitin ligase, FRQ oscillates with a period of ∼24 h despite its severely compromised turnover (44). Mutations of S781A on l-FRQ and S682A on s-FRQ showed no significant effects on the conidial period despite their confers decreased turnover rates of FRQ protein (Figs. 7, *B* and *E* and S4, *A*–*C*), which supports that the FRQ stability does not always correlate with the circadian period (44). FRQ-bound CK1a modulates the phosphorylation of FRQ and WCC; similarly, mammalian PER acts as a scaffold of CK1 to phosphorylate itself and CLOCK (19, 45, 46). The *frq7* and *frq1* strains display a longer period and a short period, respectively (47). Liu *et al.* (20) found that the binding of CK1a to FRQ7 was weaker than FRQ1, suggesting that the association affinity between FRQ and CK1a determines the circadian period length. Consistently, in this work, the *l-frq* strain with a shorter period showed a stronger CK1a-FRQ association compared to *s-frq* strain (Fig. 1*G*), and consistently, l-FRQ displayed higher phosphorylation and faster turnover rate (Figs. 2, *A*–*F* and *H* and S2, *A*–*C*). These data provide more evidence that the recruitment of CK1 by the circadian negative components acts in the positive and negative feedback loops.

The association between FRQ and FRH plays multiple important roles in regulating FRQ phosphorylation, stability, interaction with other partners, and clock function (5, 48, 49, 50). FRQ6B2 and FRQ6B5 are two subdomains located in FRQ6B, the deletion of which abolishes the FRQ-FRH interaction (37, 49). T781 and T782 are the only two threonine sites in FRQ6B2 and FRQ6B5 and mutations of these two sites showed no overt effects on the FRQ-FRH interaction (Fig. 7*D*), suggesting that the formation of the FRQ–FRH complex is independent of the phosphorylation status of FRQ.

FRQ is an intrinsically disordered protein (35, 36), and it has been proposed that FRQ might exist as a non-globule-shaped molecule (18). In *Drosophila*, phosphorylation of PER leads to a more open conformation than the hypophosphorylated isoforms, and phosphorylation decreases the stability of PER (51). Hyperphosphorylated FRQ possesses a looser conformation than hypophosphorylated species (36). Consistently, in this work, we revealed that l-FRQ might have a structure looser than s-FRQ (Fig. 3, *A* and *B*). We also observed lower phosphorylation of WCC proteins in the *l-frq* strain at 22 °C, 25 °C, and 28 °C (Fig. 1*H*), which cannot be explained by the higher l-FRQ-CK1 affinity (Fig. 1*G*). It is possible that although l-FRQ shows higher binding affinity with CK1, it may be less efficient to promote the phosphorylation of WCC due to its looser conformation. Alternatively, the phosphorylation difference in WCC proteins between s-FRQ and l-FRQ may be controlled by other kinase(s) rather than CK1. These issues remain to be investigated.

The extensive phosphorylation of FRQ occurs in a dynamic and circadian fashion, in which phosphorylation of the N-terminus of l-FRQ occurs late in the circadian cycle (17). The N-terminal portion of FRQ is positively charged while the C-terminal portion is negatively charged, and it has been proposed that the N and C parts of FRQ bind together to form an intramolecular hinge-like structure (36). However, this hinge-like structure fails to explain the late phosphorylation phase of the l-FRQ N terminus (51). Since FRQ proteins form dimers *via* their coiled-coil (CC) domains, which are critical for maintaining WCC expression and circadian rhythmicity (10), an FRQ dimer might be composed of two hinge-like FRQ proteins bound together *via* their CC domains (Fig. 7*F*). A looser conformation in the middle part of l-FRQ would facilitate the accessibility of CK1a with FRQ-CK1a interaction domains (FCDs) which might explain the late phase of phosphorylation of the N terminus (17). In contrast, the intermolecular binding between the FRQ dimer *via* the adjacent CC domains might be so compact that it probably hinders the accessibility of kinases and phosphorylation as consequence, which may explain the late phosphorylation phase of the N terminus of l-FRQ (Fig. 7*F*).

Synchronization is critical for the circadian clock. In the WT strain, FRQ proteins might exist as three forms of dimers: l-FRQ/l-FRQ, s-FRQ/s-FRQ, and s-FRQ/l-FRQ, which implies that even in one single cell, the regulation of each set of clock machinery may not be homologous. In *Drosophila* and mammals, PER and CRY/TIM proteins are the counterparts of FRQ in the circadian negative feedback loop, and PER and CRY contain paralogues in mammals (4, 52, 53). It will be interesting to investigate how the pool of different clock protein isoform dimers acts in concert to generate molecular rhythms.

## Conclusion

In this study, we provided further evidence supporting the differential regulation of phosphorylation, structure, and stability of FRQ isoforms which control the *Neurospora* circadian clock; the specific N-99 region plays a critical role in regulating the post-translational modification and function of l-FRQ. In addition, we identified the differentially phosphorylated sites on FRQ isoforms and two novel phosphorylation sites. The regulatory mechanisms of circadian clocks are highly conserved in eukaryotes. These findings will also further our understanding of the underlying regulatory mechanisms of the circadian clock in other organisms. In the future, resolving the structure of FRQ and its dimers probably through Cryo-EM will shed light on the in-depth understanding of the differences in post-translational modification, conformation, and function between FRQ isoforms.

## Experimental procedures

### *Neurospora* strains and culture

*303-3* (*bd*, *his3*, *frq*^*10*^), the *frq* null strain, is the host strain for all FRQ mutant constructs. *Pkaj120* plasmids bearing different deletions were transformed into the *frq*^*10*^ host strain at the *his-3* locus. *L-frq* and *s-frq* strains were generated by transforming plasmids harboring mutations at the first and the third initiation codons into *frq*^*10*^, respectively (21). *Pkaj120* is an *frq* null strain transformed wild-type *kaj120* plasmid as a control strain (18). The *301-5* (*bd*) strain was also used as wild-type control in indicated experiments (26). The *mdr3*^*KO*^ strain was purchased from the Fungal Genetics Stock Center (FGSC13545). To construct the *pkaj120* plasmids comprising different mutations or deletions, the mutations were first generated in partial *frq* sequence cloned in *puc19* vector by site-directed mutagenesis strategy through overlap extension using PCR with primers harboring respective mutations. Next, the fragments were subcloned into *pkaj120* through specific restriction enzyme sites.

### Race tube assay

A race tube is a long glass tube containing a layer of solid media inside, used for the determination of the circadian period of *Neurospora* in constant darkness. In race tube assay, *Neurospora* is inoculated inside the race tube which allows it to grow in one-way. During growth, *Neurospora* releases asexual conidia in a circadian fashion, and the circadian period can be calculated according to the interval time between the conidiation bands (54). The medium for race tube assays contained 1× Vogel’s medium, 2% glucose, 50 ng of biotin/ml, and 1.5% agar. After transfer into a dark room, the growth fronts were marked on the race tubes at certain time points every 24 h.

### Protein analysis and ChIP assay

Protein extraction, quantification, Western blot analysis, and immunoprecipitation assays were performed as described previously (49). For analysis of the phosphorylation of WCC proteins, specific SDS-PAGE gel (acrylamide-bisacrylamide 149:1) was prepared (13). Transfer of protein from gel to the membrane in Western blot was conducted under 120 mA for 2.5 h using the Mini-PROTEAN Tetra (Bio-Rad) apparatus. ChIP assays were performed as described previously (55). Immunoprecipitation was performed with a WC-2 antibody. The following primers were used in the ChIP assay: forward, 5′-tgtccaagcgggaagctggagt-3′; reverse, 5′-ccacgcttagggtaagtaactg-3′.

### Freeze-thaw experiments and incomplete trypsin digestion analysis

To perform protein freeze-thaw assays, the protein extracts were diluted to a concentration of 5 μg/μl, then frozen in liquid nitrogen, and thawed in a water bath at room temperature, for a specific number of cycles. The trypsin digestion assay was performed as described previously (36, 56). Briefly, 100 μl of the protein extracts (2.5 μg/μl) was treated with 50 ng trypsin (final concentration 0.5 μg/ml) at 25 °C, and the aliquots of 20 μl were taken out after trypsin addition at 0, 5, 15, 30 min. The samples were subjected to Western blot analysis.

### Protein purification and thrombin cleavage assay

The thrombin cleavage site of FRQ was predicted by the Peptidecutter online tool (https://web.expasy.org/peptide_cutter/) (57). L-FRQ-c-Myc-His and s-FRQ-c-Myc-His were expressed and enriched with anti-c-Myc beads (Sigma), which were further cleaved by thrombin (final concentration: 10 ng/μl) at 4 °C for 5 h. Subsequently, the samples were treated with λ-PPase (final concentration: 20 U/μl) by incubating at 30 °C for 30 min. The cleaved protein was subject to Western blot analysis using FRQ antibody.

### Protein purification and label-free quantitative LC/MS analysis of s/l-FRQ

Label-free quantitative LC/MS analysis was used to identify FRQ peptides with differential phosphorylation patterns (58). The label-free LC/MS experiments were conducted by Novogene Co, LTD.

### Statistical analysis

Data are the mean values ± SD or mean values ± SE as indicated. Experimental data were analyzed using Student *t* test in Microsoft Excel software. A *p*-value of <0.05 was considered significant. ∗*p* < 0.05, ∗∗*p* < 0.01, ^#^*p* < 0.001.

## Data availability

All data in this study are available within the article, supporting information, and/or from the corresponding author on reasonable request.

## Supporting information

This article contains supporting information.

## Conflict of interest

The authors declare that they have no conflicts of interest with the contents of this article.
