# Supplementary material for: Differential regulation of phosphorylation, structure, and stability of circadian clock protein FRQ isoforms
Source: J Biol Chem. 2023 Mar 9;299(4):104597. doi: 10.1016/j.jbc.2023.104597 (PMC10140173; doi:10.1016/j.jbc.2023.104597)
Supplement: Supporting Figures S1–S4 and Table S1 [file mmc1.pdf]

Supplemental Information

Differential regulation of phosphorylation, structure and stability of FRQ isoforms which control *Neurospora* circadian clock

Xihui Gan, Xianyun Chen, Xiaolan Liu, Huan Ma, Lin Zhang, Peiliang Wang, Yunzhen Lin, Xiaoling Yang, Ling Fang, Tianyu Huang, Yingying Liang, Xiaolan Liu, Xiao Liu, Jinhu Guo

Table of contents

1. Supplementary Figure 1
2. Supplementary Figure 2
3. Supplementary Figure 3
4. Supplementary Figure 4
5. Supplementary Table 1

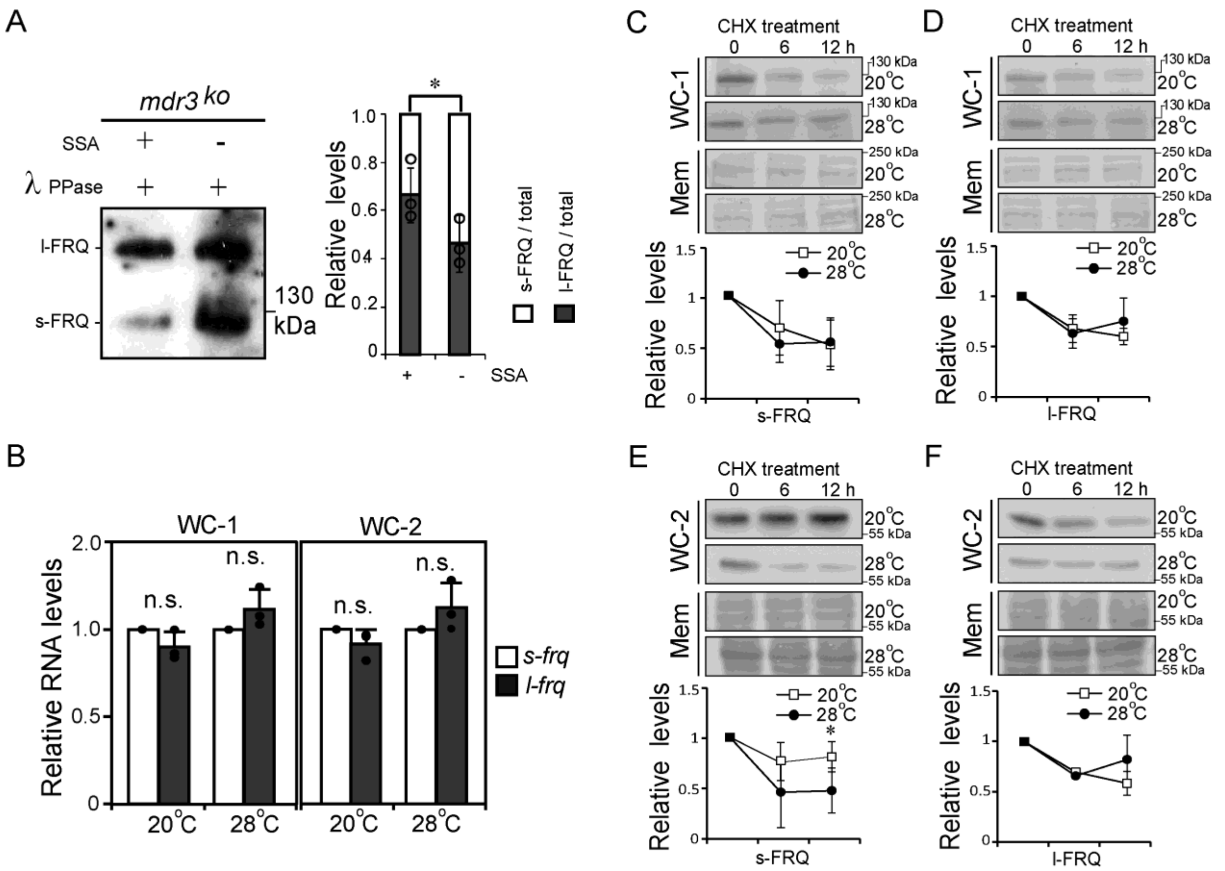

Fig. S1 Characterization of *Neurospora* FRQ isoforms l-FRQ and s-FRQ. ***A*****,** Repression of s-FRQ production by SSA. λ-phosphatase was added to dephosphorylate the protein samples. Statistical results are shown on the right. ***B*****,** qRT-PCR results revealed no significant differences in the expression of *frq* transcripts in *s-frq* and *l-frq* strains at indicated temperatures. n.s., non-significant. ***C-F*****,** Western blot results showing degradation of s-FRQ and l-FRQ after CHX treatment (10μg/ml) at three temperatures. Statistical results are shown on the bottom panels. Data are the mean ± SD. n = 3. *, $P < 0.05$.

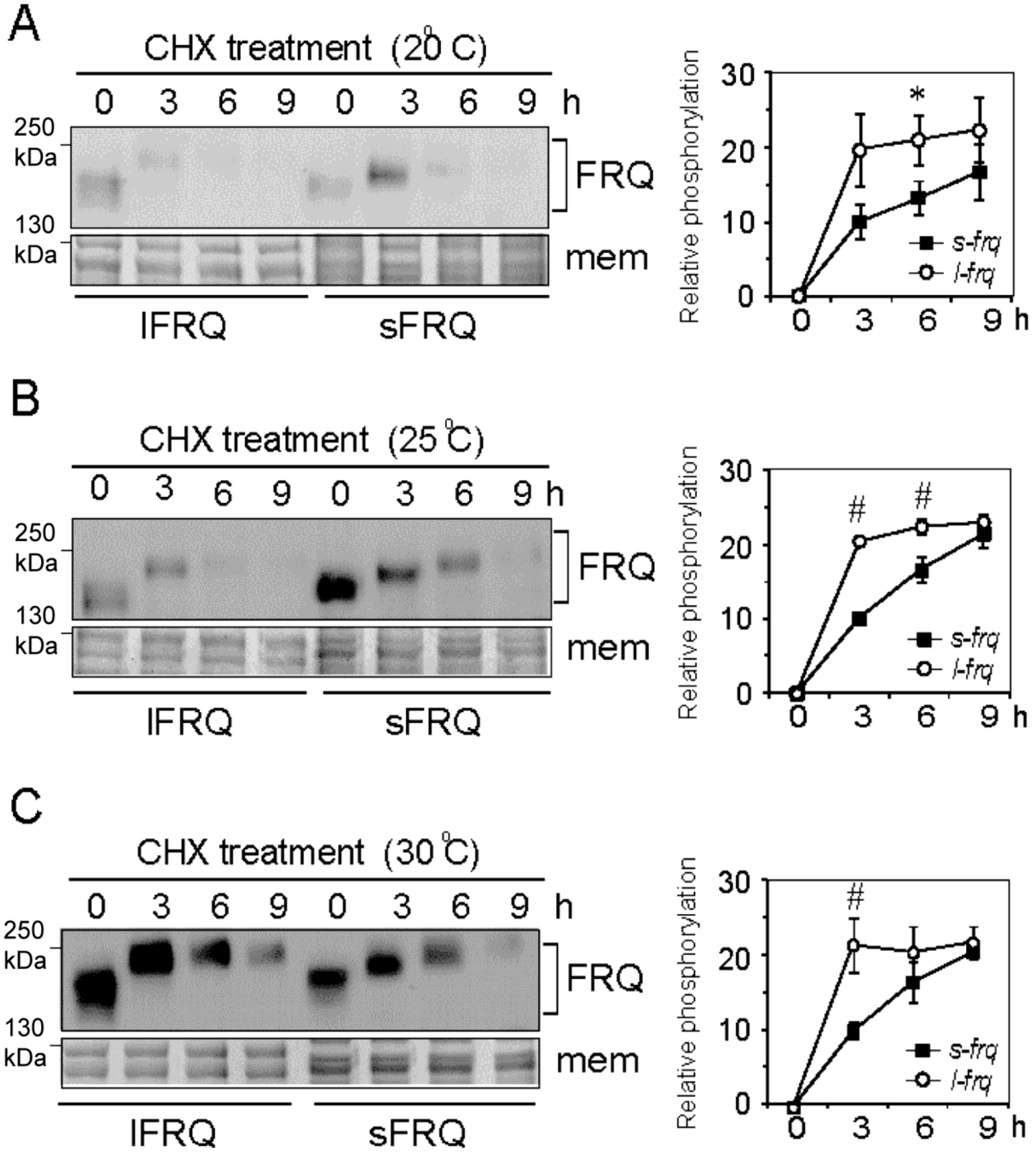

Figure S2. CHX treatment leads to more rapid phosphorylation of l-FRQ than s-FRQ. ***A-C***, Western blotting analysis of s-FRQ and l-FRQ proteins from s-*frq* and l-*frq* strains, respectively. The samples were harvested after treatment with cycloheximide (CHX) at indicated time pointes. Left in each panel: the representative western blot results. Right in each panel: densitometric analysis of electrophoresis rates of s-FRQ and l-FRQ according to the western blotting results. Data are the mean values ± SE (n = 3 biological replicates), * $P < 0.05$, # $P < 0.001$.

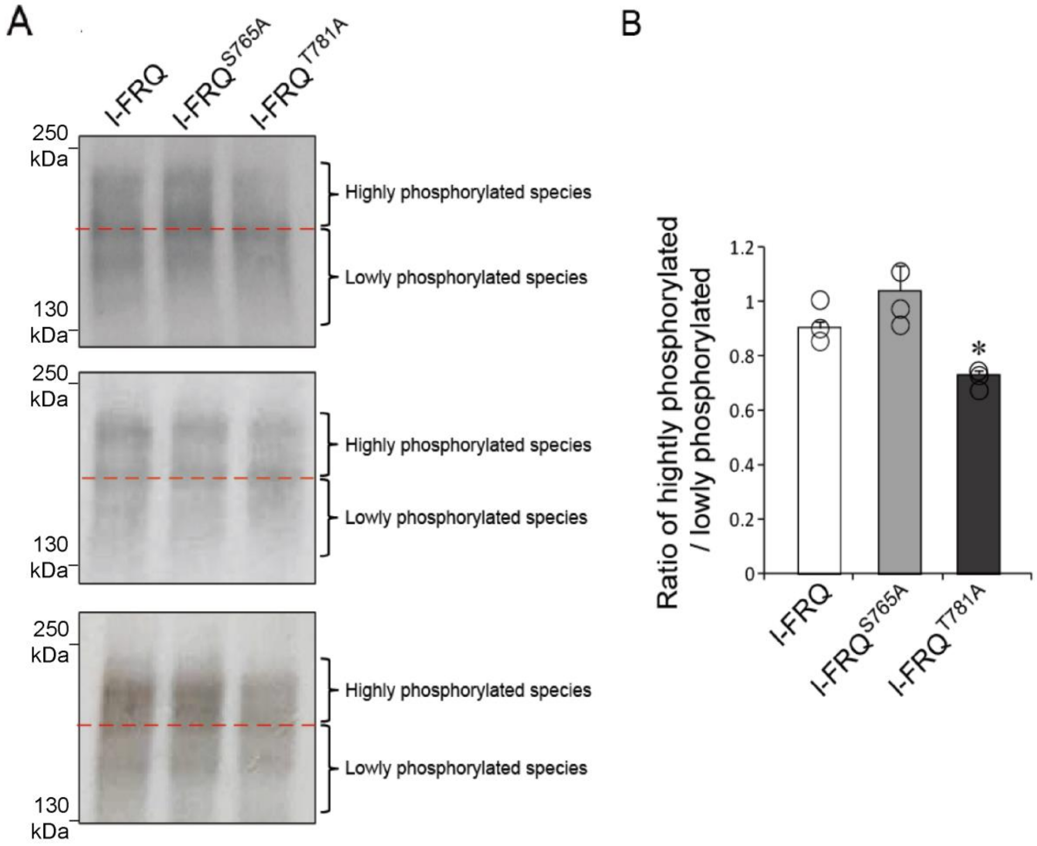

Figure S3. Comparison of FRQ phosphorylation levels. ***A,*** Western blotting results of l-FRQ and indicated mutants. In the results, FRQ exhibits smeared pattern due to extensive phosphorylation of different sites and roughly there are two dark bands in the smear. ***B,*** Statistic results of the phosphorylation ratio. The three pictures on the left were scanned and the densitometric values measured from the upper and bottom parts separated from the lower dark band on each lane (broken line in red), respectively. Subsequently the ratios of densitometric values between the upper and bottom parts of each lane were calculated. Note that the western blotting results in the top picture in **(*A*)** are also present in Figure 7C. Data are the mean values ± SE (n = 3, biological replicates), * $P < 0.05$.

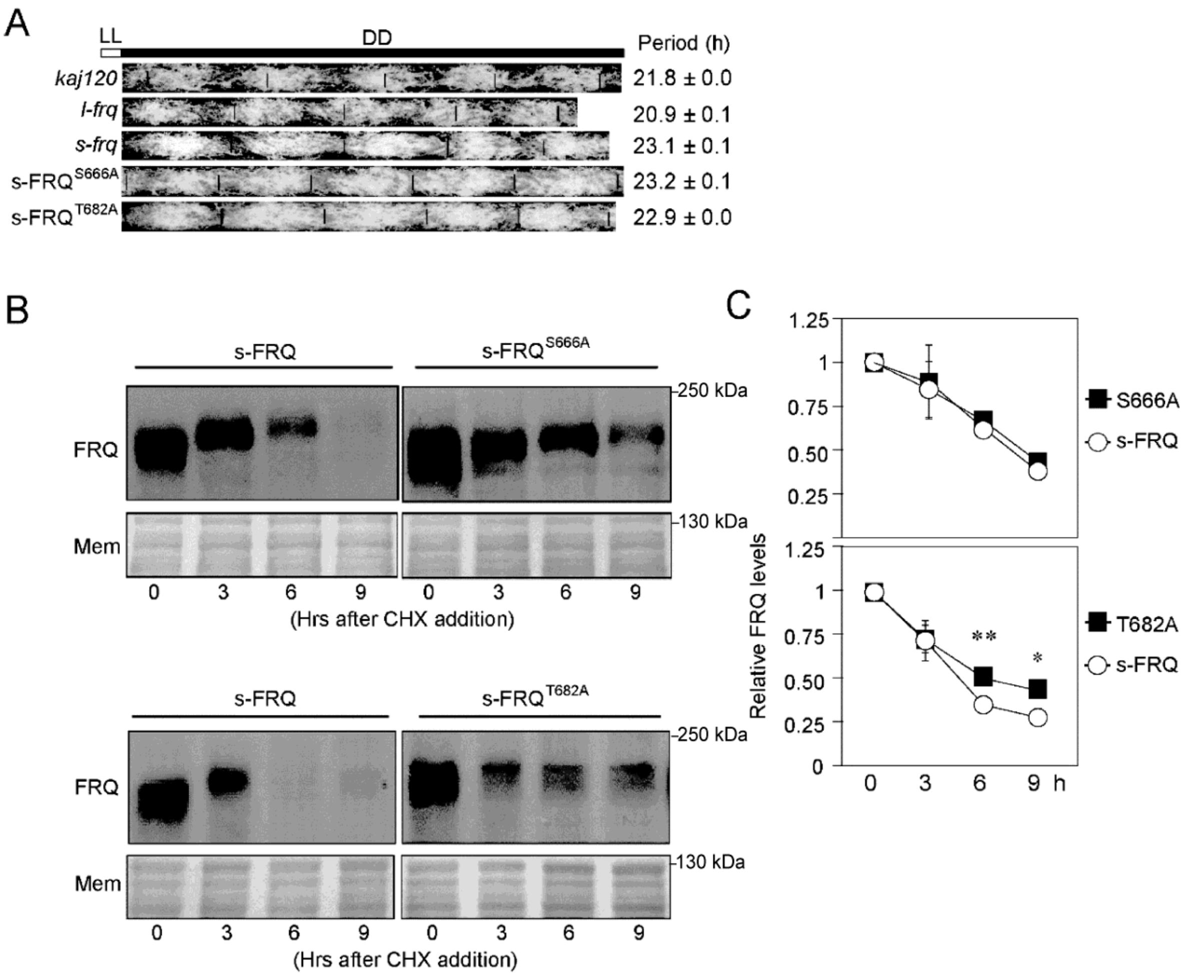

Figure S4. Characterization of novel FRQ phosphorylation sites. ***A,*** Race tube assay showing the circadian conidiation rhythms of the indicated strains. Periods of circadian are denoted on the right. *Kaj120* was used as a control. Values are the mean ± SD (n = 3). ***B,*** Western blots showing protein degradation after CHX treatment of s-FRQ^{S666A} and s-FRQ^{T682A}. Due to the 99-aa N-terminus of l-FRQ, FRQ^{S765A} and FRQ^{T781A} were mutated at the same residues to l-FRQ^{S765A} and l-FRQ^{T781A}, respectively. ***C,*** Densitometric analysis results are shown. Data are the mean ± SD (n = 3). *, $P < 0.05$ and **, $P < 0.01$.

Table S1: Differentially phosphorylated peptides in s-FRQ and l-FRQ.

Sequence	Modifications	Abundances (Normalized) in s-FRQ			Abundances (Normalized) in l-FRQ			average		*P* values
		Sample #1	Sample #2	Sample #3	Sample #1	Sample #2	Sample #3	s-FRQ	l-FRQ	
AEAVDGDDSPLAAVTGK	1xPhospho [S9]	5130444	9609056.821	9313655.842	20670252.8	17332275.9	7197242.5	8017719	15066590	0.088302813
EVMEPSGLGGVLPDDHFVMLVTTR	1xPhospho [T/S]	1936185.532	0	0	0	0	0	645395.2	0	0.186950483
NQSMPSMDAPLAPEGTNMAPPRPPPEGLR	1xPhospho [S3]	374276.875	474903.0878	711148.3925	1360278.65	1056404.26	629512.875	520109.5	1015399	0.051012331
QLSRSTTSEDTAEFIAER	1xPhospho [T/S]	185512.5469	115836.9372	0	0	0	0	100449.8	0	0.068465147
STTSEDTAEFIAER	1xPhospho [T/S]	2066873.344	3023403.037	3632442.675	6867579.52	4584842.49	2467312.88	2907573	4639912	0.13432528
WDFSGSGTTQQR	1xPhospho [S6]	350693.6236	395742.2328	1494075.749	994478.373	524019.531	0	746837.2	506166	0.318291002
TLSGSSLPIRPLSDDR	2xPhospho [S/T]	1793831.5	2308460.278	1417600.727	0	0	0	1839964	0	0.001025061
DNGSASNSGGDQTELGGTGTGSGDGSGSGGR	1xPhospho [T/S]	0	0	0	900166.519	690348.194	205092.422	0	598535.7	0.021886311
RASPDKSITLENHR	1xPhospho [S/T]	0	0	0	5493008.76	5423381.98	4455973.81	0	5124122	5.30763E-05
RASPDKSITLENHR	2xPhospho [S3; S7]	0	0	0	738564.586	474617.081	288351.875	0	500511.2	0.00929195
VTSSSALGVTESQPQLK	1xPhospho [T/S]	0	0	0	1716555.18	1167822.88	579427.313	0	1154602	0.012262381
VTSSSALGVTESQPQLKSSPTR	1xPhospho [S/T]	0	0	0	1399325.22	1006502.77	1135908.88	0	1180579	0.000258804
RNSSGESEPTNWFNQSNR	1xPhospho [S]	0	0	0	18090701.7	15677280.2	11754692.5	0	15174225	0.00059728
AKSVGTQKVENYLR	1xPhospho [S3]	0	0	0	709089.469	689456.309	1456461.81	0	951669.2	0.009805752
AKSVGTQKVENYLR	1xPhospho [S/T/Y]	678329.375	960870.6317	629223.6358	0	0	0	756141.2	0	0.00092806
SVGTQKVENYLR	1xPhospho [S1]	287339.625	392808.6138	268901.5848	439208.792	418081.039	730681.188	316349.9	529323.7	0.059944214
TGNNTSPPGAIAPDQRPTRPR	1xPhospho [S/T]	0	0	0	4713762.94	3824183.05	3112539.47	0	3883495	0.000553528
TGNNTSPPGAIAPDQRPTRPR	1xPhospho [T/S]	590067.0508	835718.6209	1568227.545	0	0	0	998004.4	0	0.013677611
SPSDTFHYKPMFVHR	1xPhospho [Y/S/T]	0	0	0	136146.381	435469.948	398452.469	0	323356.3	0.013241273
FGPSRSPSDTFHYKPMFVHR	1xPhospho [S/Y/T]	2013119	1473089.805	4180629.933	0	0	0	2555613	0	0.01830505
SPSDTFHYKPMFVHR	1xPhospho [S/T/Y]	3370355.188	1130249.06	2485978.342	0	0	0	2328861	0	0.011635012
FGPSRSPSDTFHYKPMFVHR	1xPhospho [S/Y/T]	2013119	1473089.805	4180629.933	0	0	0	2555613	0	0.01830505
SPSDTFHYKPMFVHR	1xPhospho [S/T/Y]	3370355.188	1130249.06	2485978.342	0	0	0	2328861	0	0.011635012
EVEGSGSGDEVEHVLQR	1xPhospho [S]	9603787.688	15988595.32	20242705.2	31622313.4	24878822.5	15774135.6	15278363	24091757	0.093287585
TLSGSSLPIRPLSDDRAR	1xPhospho [T/S]	0	0	0	399212.581	653392.841	1392067.63	0	814891	0.026042624
TLSGSSLPIRPLSDDR	2xPhospho [S/T]	1793831.5	2308460.278	1417600.727	0	0	0	1839964	0	0.001025061
TSSPLPPR	1xPhospho [T/S]	0	0	0	651084.289	536232.389	109919.398	0	432412	0.029192815
TSSPLPPR	1xPhospho [S/T]	43966.82813	82917.20917	80644.95178	0	0	0	69176.33	0	0.002698064

Same amount of purified s-FRQ and m-FRQ was subject to label-free LC/MS analysis. The levels of phorphorylated peptides derived from digestion are shown. The results comprise independent triple replicates for each protein. Student's t test was performed to calculate *P* values of quantity of these differentially phosphorylated peptides. $P < 0.05$ denotes statistical significance. The phosphorylated peptides with *P* values > 0.05 are not shown.
